# Vitamin D and Lipid Profiles in Postmenopausal Women: A Meta-Analysis and Systematic Review of Randomized Controlled Trials

**DOI:** 10.3389/fmolb.2021.799934

**Published:** 2021-12-17

**Authors:** Weiting Liu, Zezhen Wu, Dan Zhu, Genben Chen, Guiming Yan, Shuo Zhang, Fengwu Chen, Barkat Ali Khan, Kaijian Hou

**Affiliations:** ^1^ School of Nursing, Anhui University of Chinese Medicine, Hefei, China; ^2^ The First Affiliated Hospital of Shantou University Medical College, Shantou, China; ^3^ Department of Endocrine and Metabolic Diseases, Longhu People’s Hospital, Shantou, China; ^4^ Department of Endocrine and Metabolic Diseases, Chaoan District People’s Hospital, Chaozhou, China; ^5^ Department of Endocrine and Metabolic Diseases, the First Affiliated Hospital of Medical College of Shantou University, Shantou, China; ^6^ Drug Delivery and Cosmetics Lab (DDCL), GCPS, Faculty of Pharmacy, Gomal University, Dera Ismail Khan, Pakistan

**Keywords:** vitamin D, lipid profile, HDL, LDL, triglycerides

## Abstract

**Background and Aim:** It is known that hyperlipidemia and low vitamin D level are risk factors associated with cardiovascular disease (CVD). However, the effect of vitamin D administration on lipid profiles in postmenopausal women remains unclear. This study aims to evaluate the effect of vitamin D on lipid profiles in postmenopausal women based on meta-analysis and systemic review.

**Methods:** The literature search was performed in multiple databases (Scopus, PubMed/Medline, Web of Science, and Embase) from 1997 to 2021. The statistical analysis was performed using the Stata software version 14 (Stata Corp. College Station, Texas, United States). The effects of vitamin D administration of the lipid profiles, including Triacylglycerol (TG), LDL-Cholesterol (LDL-C), HDL-Cholesterol (HDL-C), and Total Cholesterol (TC) were evaluated by the Der Simonian and Laird random effects model. The weighted mean difference (WMD) and 95% confidence intervals (CI) were calculated.

**Results:** The level of TG changed significantly by −3.76 mg/dl (CI: −6.12 to −1.39, *p* = 0.004) and HDL-C by 0.48 mg/dl (CI: −0.80 to −0.15, *p* = 0.004) in vitamin D administration group [11 eligible trials (placebo = 505 participants, vitamin D intervention = 604 participants)] compared to the control group in the postmenopausal women. Taking into account this comparison between groups, in contrast, the level of LDL-Cholesterol (LDL-C) (WMD: 0.73 mg/dl, 95% CI: −1.88, 3.36, *p* = 0.583) and TC (WMD: 0.689 mg/dl, CI: −3.059 to 4.438, *p* = 0.719) did not change significantly.

**Conclusion:** In conclusion, the vitamin D administration in postmenopausal women, decreased the concentrations of TG, and HDL-C, but have no effects on LDL-C and TC.

## Introduction

Cardiovascular disease (CVD) is a common disease of the circulatory system, which is closely associated with atherosclerosis (Centers for Disease and Prevention 2011; Damorou et al., 2014). Vascular lesions caused by CVD are the main causes of premature and sudden death in human beings (Levenson et al., 2002). The incidence of CVD increases with age, and it is estimated that by 2030, 23.6 million people will die from cardiovascular disease each year (Damorou et al., 2014). CVD poses a huge threat to human health. There are many causes of CVD, including hypertension (HTN), obesity, hyperlipidemia and diabetes. Recently, vitamin D deficiency is increasingly being recognized as a potential cardiovascular risk factor. Low levels of vitamin D are associated with the development of cardiovascular risk factors such as hypertension, obesity, hyperlipidemia, and diabetes (Forman et al., 2007; Martins et al., 2007; Knekt et al., 2008; Jorde et al., 2010).

Vitamin D is a hormone synthesized in the skin in response to exposure to ultraviolet B or sunlight. The vitamin D synthetic process includes the formation of cholecalciferol of cholesterol in the body and the formation of 25-hydroxyvitamin D 25(OH) D by the hydroxylation in the liver. The 25(OH) D is further hydrogenated into 1,25-dihydroxyvitamin D [1,25(OH)2 D] by the kidneys, and the liver and kidneys play critical roles in vitamin D synthesis (Elsori and Hammoud 2018). Vitamin D is essential because it is synthesized in the body and binds to receptors to perform its functions similar to other hormones. The main role of vitamin D is to promote bone mineralization, maintain healthy bone structure and maintain serum calcium and phosphate concentrations in regulating the interaction between osteoblasts and osteoclasts. In addition, vitamin D can inhibit cell proliferation, induce terminal differentiation, inhibit angiogenesis, induce insulin production, and inhibit renin production (Rosen 2011). In addition, vitamin D regulates immune function, cell growth, and neuromuscular activity and reduces inflammation. Vitamin D deficiency has been associated with cardiovascular disease, diabetes, cancer, and more (Pittas et al., 2010). Several experimental studies in animals and cell cultures have shown that vitamin D receptor activation protects against cardiovascular disease (Pludowski et al., 2013). In 2014, a systematic review and meta-analysis conducted by Chowdhury et al. described similar results, suggesting that 25(OH) D was inversely associated with the risk of death from cardiovascular disease and other causes (Chowdhury et al., 2014). Vitamin D deficiency was linked to a high incidence of cardiovascular events in a prospective observational study (Zittermann and Prokop 2014).

Perimenopausal and postmenopausal women are at a risk of VD deficiency as aging and increased fat mass lead to decreased blood vitamin D level. In addition, appropriate physical activity and Sun exposure are necessary for the synthesis of vitamin D in the skin (Pludowski et al., 2013), and the amount of vitamin D in the body decreases with age, especially at menopause (Group 2011). The presence of a range of cardiovascular risk factors such as insulin resistance, obesity, atherosclerotic dyslipidemia, and hypertension is known as metabolic syndrome. Metabolic syndrome is one of the major health problems in postmenopausal women and a major cause of cardiovascular morbidity (CVD) and mortality in this group of women (Perk et al., 2013; Yasein et al., 2015). Typically, the age of first onset of atherosclerotic coronary heart disease in women is 9–10 years later than in men due to estrogen (Anand et al., 2008), and the ovarian hormone concentrations decline in menopausal transition. Therefore, part of the cause of CVD in perimenopause and menopause is related to the decline in ovarian hormone concentrations during and after menopause (Atsma et al., 2006). In addition, estrogen deficiency is also associated with the risk of central obesity, which increases the risk factors and prevalence of cardiovascular disease after menopause (Lovejoy 2003; Lobo 2008). Previous reports suggest that the prevalence of metabolic syndrome in postmenopausal women may be as high as 41.5% (Chedraui et al., 2007; Ding et al., 2007). In 2012, Wang et al. found that low levels of vitamin D were associated with cardiovascular disease (Wang et al., 2012). Dyslipidemia is a major risk factor for CVD and atherosclerosis (Polkowska et al., 2015). Epidemiological studies speculate that there may be a negative association between vitamin D status and metabolic syndrome (Ford et al., 2005; Reis et al., 2008; Lee et al., 2009). Vitamin D deficiency is associated with hyperlipidemia. However, the exact effect of vitamin D on lipid profiles in postmenopausal women is unclear.

### Rational and Limitations

In this study, we evaluate the effect of vitamin D on lipid profiles in postmenopausal women through a meta-analysis and systematic review. The main rational to conduct a systematic review is the benefit of collating evidence from a variety of sources while the limitations include the risk of bias.

## Methodology

This meta-analysis was conducted following the Preferred Reporting Items for Systematic Reviews and Meta-analyses (PRISMA) guidelines (Moher et al., 2009).

### Search Strategy

We developed a sensitive search strategy for multiple databases (Scopus, PubMed/Medline, Web of Science, and Embase) from 1997 to May 1, 2021 (Supplementary Table S1). We did not apply language or time restrictions. We reviewed the reference lists of all the included trials for potentially eligible publications.

### Inclusion Criteria and Exclusion Criteria

To be included in the meta-analysis, 1) the trials had to be conducted on postmenopausal women, to be designed as RCTs and the intervention group had received vitamin D. 2) the trials needed to compare supplementation with vitamin D in postmenopausal women versus a control group and report the mean and standard deviation (SD) for one of the following outcomes at the end of intervention and at the beginning of the RCT in order to compute changes from the baseline: Triacylglycerol (TG), HDL-Cholesterol (HDL-C), LDL-Cholesterol (LDL-C), and Total Cholesterol (TC).

The exclusion criteria were the following: 1) editorials, non-research letters, reviews; 2) lack of a control group; 3) the data on the lipid profiles were not available.

### Data Extraction

The data were extracted independently by two reviewers. We extracted the publication and trial details (first author’s name, sample size and number of participants in each study group, mean age, treatment duration, study country, study design, vitamin D dosage, year of publish), relevant outcome information (mean and SD of TG, HDL-C, TC, LDL-C), population characteristics (health status). The data were extracted independently by two authors and any disagreements were resolved by consensus with the senior author.

### Quality Assessment

We used the Cochrane collaboration’s tool to assess the risk of bias for each trial outcome based on the following domains: 1) deviations from the intended interventions, 2) the randomization process, 3) the outcome measurement, 4) missing outcome data, 5) the selection of the reported results (Higgins et al., 2011).

### Statistical Analysis

The statistical analysis was performed using the Stata software version 14 (Stata Corp. College Station, Texas, United States). The Der Simonian and Laird random effects model was applied and the weighted mean difference (WMD) and 95% confidence intervals (CI) were calculated for the vitamin D administration groups and the control groups using combined estimates (Hozo et al., 2005; Higgins et al., 2011). When the SD of the modification was not available, we derived it using the following formula: SD differences = square root [(SD baseline2 + SD end2)–(2 × R × SD baseline × SD end)]. The heterogeneity of the results of the pooled trials was checked by the I2 test levels and a *p*-value of less than 0.10. We used subgroup analyses to track potential sources of heterogeneity among the trials. The publication bias was assessed *via* the Egger test and the visual inspection of the funnel plots. In the sensitivity analyses, we excluded each study and recalculated the combined estimates to detect studies with a high-risk of bias (Egger, Davey Smith et al., 1997). If publication bias was detected, we used the trim-and-fill test to estimate negative unpublished trials and to amend the combined estimates (Duval and Tweedie 2000).

## Results

### Study Selection and Characteristics of the Included Studies

**FIGURE 1:**
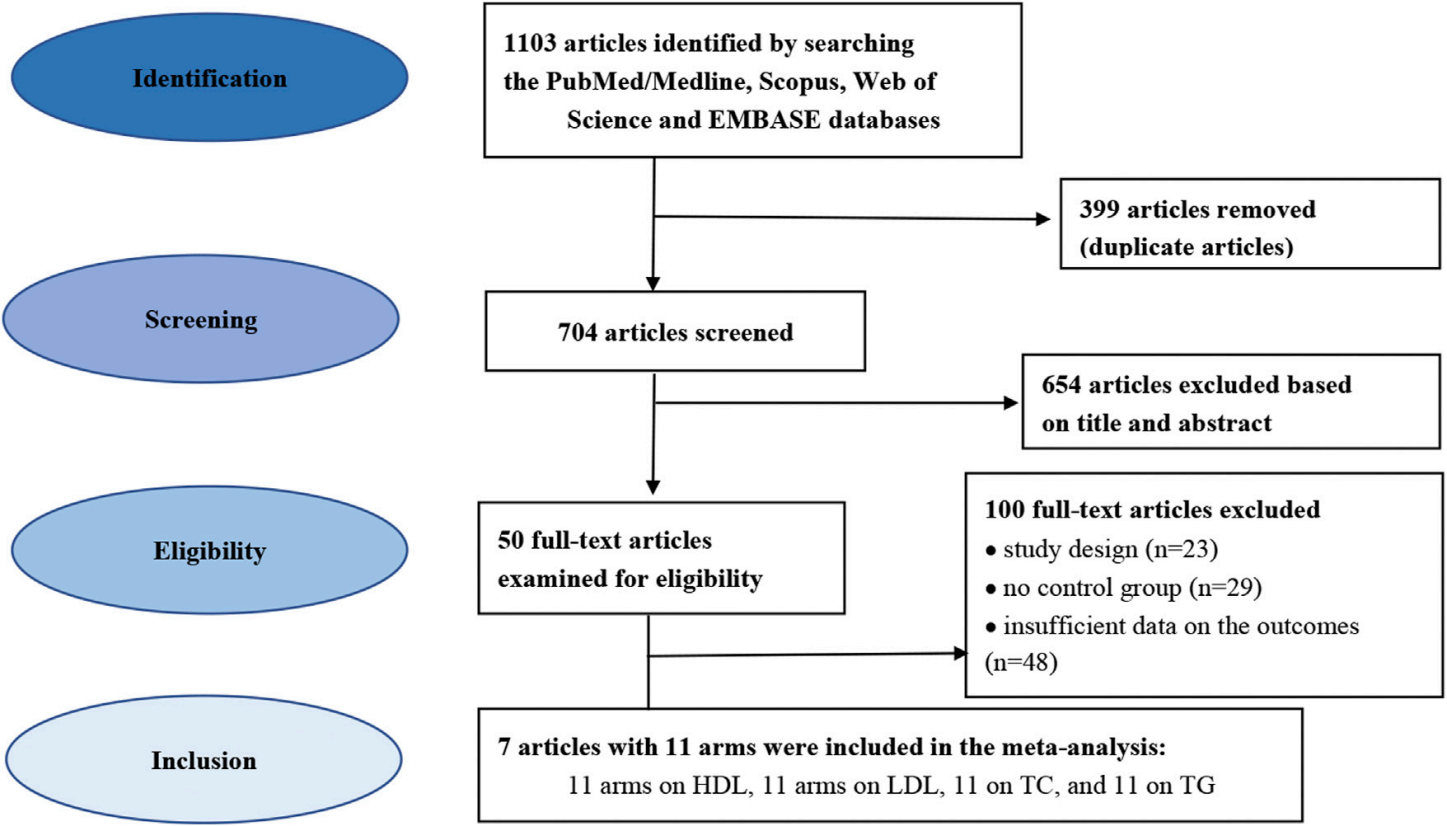
Flowchart depicting the study selection and inclusion process for the present meta-analysis.

The database search identified 1103 potentially relevant trials. Figure 1 illustrates the flow diagram of the literature search process. In total, 399 articles were removed as duplicates and 704 publications were excluded based on the screening of titles and abstracts, and the full texts of 50 trials were selected for further examination. Finally, 7 eligible trials were included in the final quantitative analyses based on the exclusion and inclusion criteria. These trials consisted of 11 arms on LDL-C, 11 arms on HDL-C, 11 arms on TC, and 11 arms on TG (Heikkinen et al., 1997; Wood et al., 2012; Moghassemi and Marjani 2014; Munoz-Aguirre et al., 2015; Bislev, Langagergaard Rodbro et al., 2018; Ferreira et al., 2020; Kerksick et al., 2020). Table 1 presents the characteristics of the included articles. The articles were published between 1997 and 2020, and were conducted in Brazil, Finland, Mexico, Iran, the United States of America (USA), the United Kingdom and Denmark.

**TABLE 1:** Characteristics of the eligible studies.

Author, year	Country	Population	Participant s age (years)	Sample size: tibolone/placebo	Duration	Baseline BMI(kg/m2)	Outcome	Vitamin D dose (IU/day)
Kerksick.et al. (2020) (a) (CTL = no diet + exercise control)	USA	Healthy postmenopausal women	56.1	11/9	14 weeks	31.8	LDL-C, TC, HDL-C, TG	400 (calcium with Vitamin D)
Kerksick.et al. (2020) (b) (LCHC = low calorie high carbohydrate diet)	USA	Healthy postmenopausal women	54.4	15/20	14 weeks	32.6	LDL-C, TC, HDL-C, TG	400 (calcium with Vitamin D)
Kerksick.et al. (2020) (c) (LCHP = low calorie high protein diet)	USA	Healthy postmenopausal women	52.4	16/18	14 weeks	36.4	LDL-C, TC, HDL-C, TG	400 (calcium with Vitamin D)
Ferreira et al. (2020)	Brazil	Healthy postmenopausal women	58.8	80/69	9 months	29.4	LDL-C, TC, HDL-C, TG	1000
Bislev et al. (2018)	Denmark	Healthy postmenopausal	65	40/41	12 weeks	27	LDL-C, HDL-C, TG	2800
Munoz-Aguirre et al. (2015)	Mexico	Healthy postmenopausal women	43.55	52/52	6 months	30.8	LDL-C, TC, HDL-C, TG	4000
Moghassemi and Marjani. (2014)	Iran	Healthy postmenopausal women	52.73	38/36	12 weeks	29.98	LDL-C, TC, HDL-C, TG	2000
Wood et al. (2012) (a)	United Kingdom	Healthy postmenopausal women	63.5	97/50	1 year	26.6	LDL-C, TC, HDL-C, TG	400
Wood et al. (2012) (b)	United Kingdom	Healthy postmenopausal women	64.1	95/50	1 year	26.8	LDL-C, TC, HDL-C, TG	1000
Heikkinen et al. (1997) (a)	Finland	Healthy postmenopausal women	52.8	83/95	3 years	26.8	LDL-C, TC, HDL-C, TG	300
Heikkinen et al. (1997) (b)	Finland	Healthy postmenopausal women during postmenopausal hormone replacement therapy	52.4	77/65	3 years	26.4	LDL-C, TC, HDL-C, TG	300

Note: HDL-C, high-density lipoprotein cholesterol; LDL-C, low-density lipoprotein cholesterol; TC, total cholesterol; TG, serum triglycerides; mg/d, milligrams per day; USA, United States of America.

In these trials, the duration of vitamin D administration ranged from 12 weeks to 3 years. The mean age of the participants was 54.4 years. The daily recommended dosage of vitamin D was between 300 and 4000 IU/day. The participants were premenopausal women and postmenopausal women with diabetes.

### Findings From the Meta-Analysis

#### Effects of Vitamin D on LDL-C Levels

**FIGURE 2:**
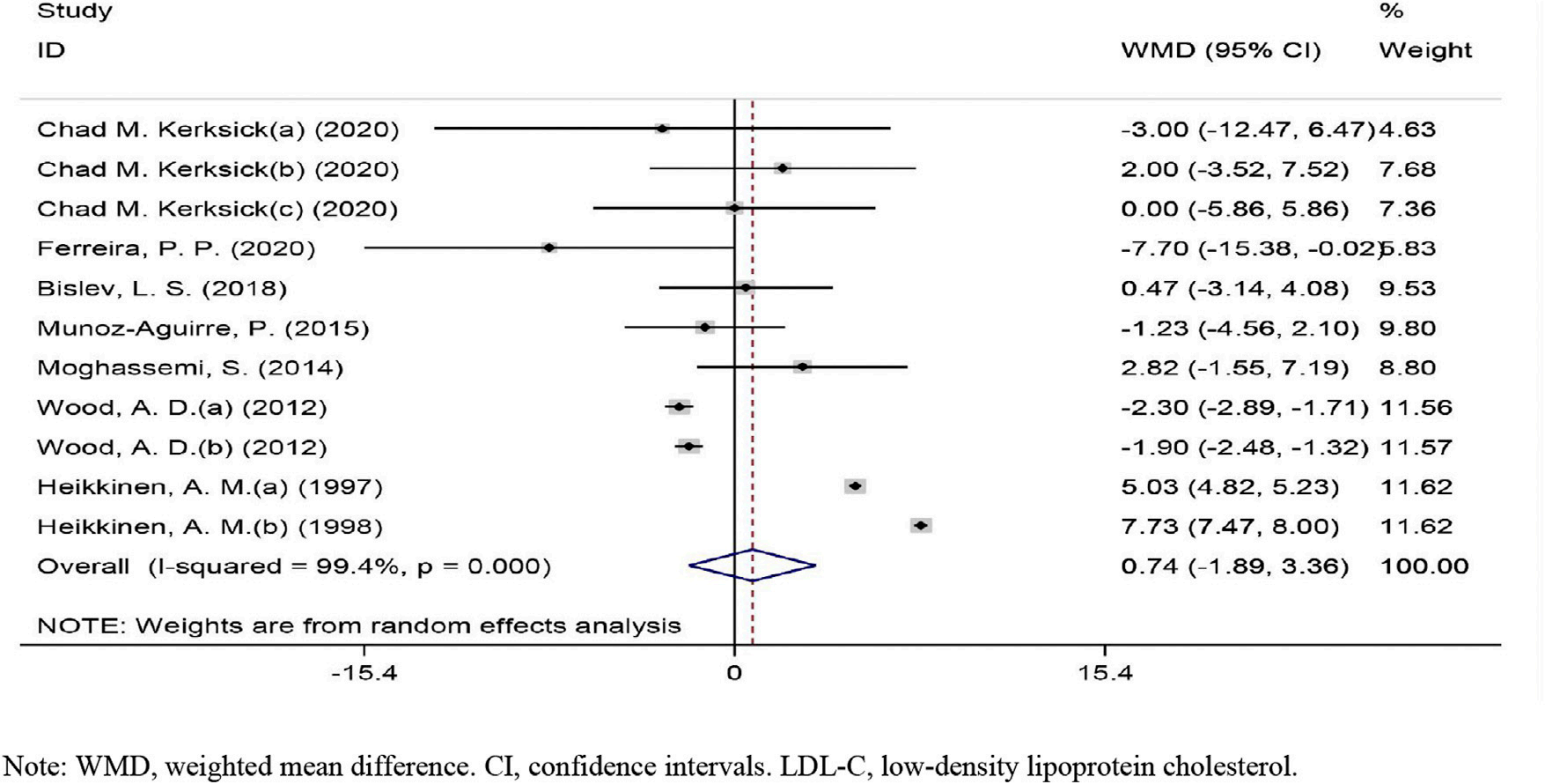
Forest plot of the randomized controlled trials investigating the effects of vitamin D on LDL-C.

The overall effect of vitamin D intervention on LDL-C level from 11 eligible trials (placebo = 505 participants, vitamin D intervention = 604 participants) are reported in Figure 2. The vitamin D intervention produced a non-significant reduction of LDL-C level at 0.73 mg/dl (CI: −1.88 to 3.36, *p* = 0.583), with a significant heterogeneity among the examined studies (I2 = 99%, *p* = 0.000). The subgroup analyses did not identify a significant impact of vitamin D supplementation on LDL-C concentrations (Supplementary Figure S1).

### Effects of Vitamin D on HDL-C Levels

**FIGURE 3:**
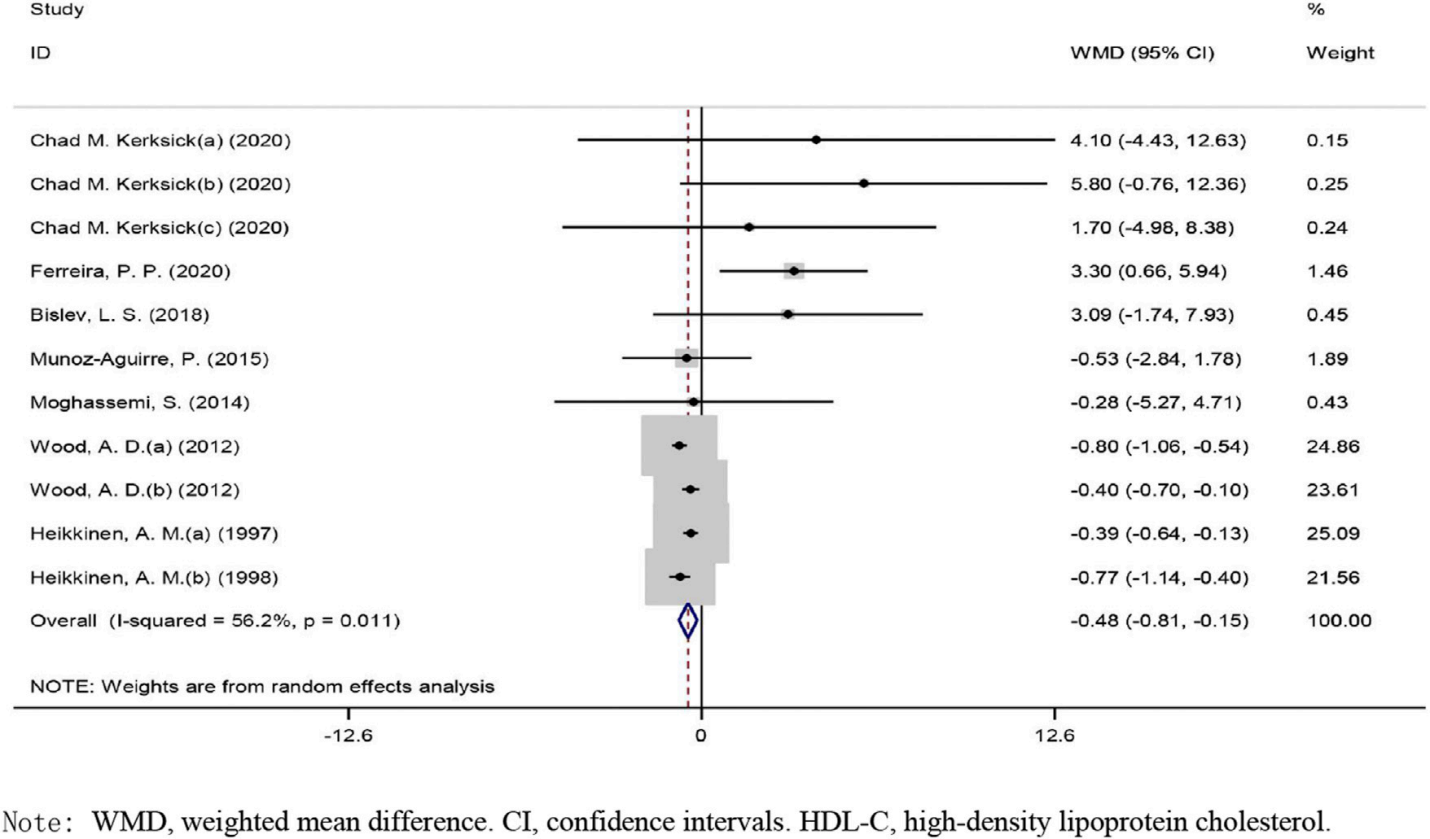
Forest plot of the randomized controlled trials investigating the effects of vitamin D administration on HDL-C.

The overall effect of vitamin D intervention on HDL-C level from 11 eligible trials (placebo = 505 participants, vitamin D intervention = 604 participants) are reported in Figure 3. The supplementation with vitamin D treatment produced a significant reduction of HDL-C level at −0.48 mg/dl (CI: −0.80 to −0.15, *p*), with a significant heterogeneity noted among the examined studies (I2 = 56%, *p* = 0.011) (Figure 3). Vitamin D reduced HDL-C in a notable fashion when the dose was ≥400 IU/day (WMD: −0.61 mg/dl, 95% CI: −0.95 to −0.28, *p* = 0.004) as compared to ˂400 IU/day (WMD: 0.63 mg/dl, 95% CI: −1.01, 2.27, *p* = 0.453) (Supplementary Figure S1). Moreover, a significant decrease was observed when the administration of vitamin D was ≥26 weeks (WMD: −0.534 mg/dl, 95% CI: −0.84 to −0.22, *p* = 0.001) versus ˂26 weeks (WMD: 2.46 mg/dl, 95% CI: −0.19, 5.11, *p* = 0.069). Based on the results of the stratified analysis, vitamin D supplementation resulted in a more pronounced reduction in HDL-C concentrations when the HDL-C baseline value was ≥50 mg/dl (WMD: −0.46 mg/dl, 95% CI: −0.82 to −0.11, *p* = 0.009) versus ˂50 mg/dl (WMD: −0.48 mg/dl, 95% CI: −2.58 to 1.60, *p* = 0.64). In addition, a notable reduction in HDL-C levels was detected in the subjects with a BMI of 25.0–29.9 kg/m2 (WMD: −0.513 mg/dl, 95% CI: −0.83 to −0.19, *p* = 0.002) as compared to subjects with a BMI ≥30 kg/m2 (WMD: 1.39 mg/dl, 95% CI: −1.61, 4.40, *p* = 0.362).

#### Effects of Vitamin D on TG Levels

**FIGURE 4:**
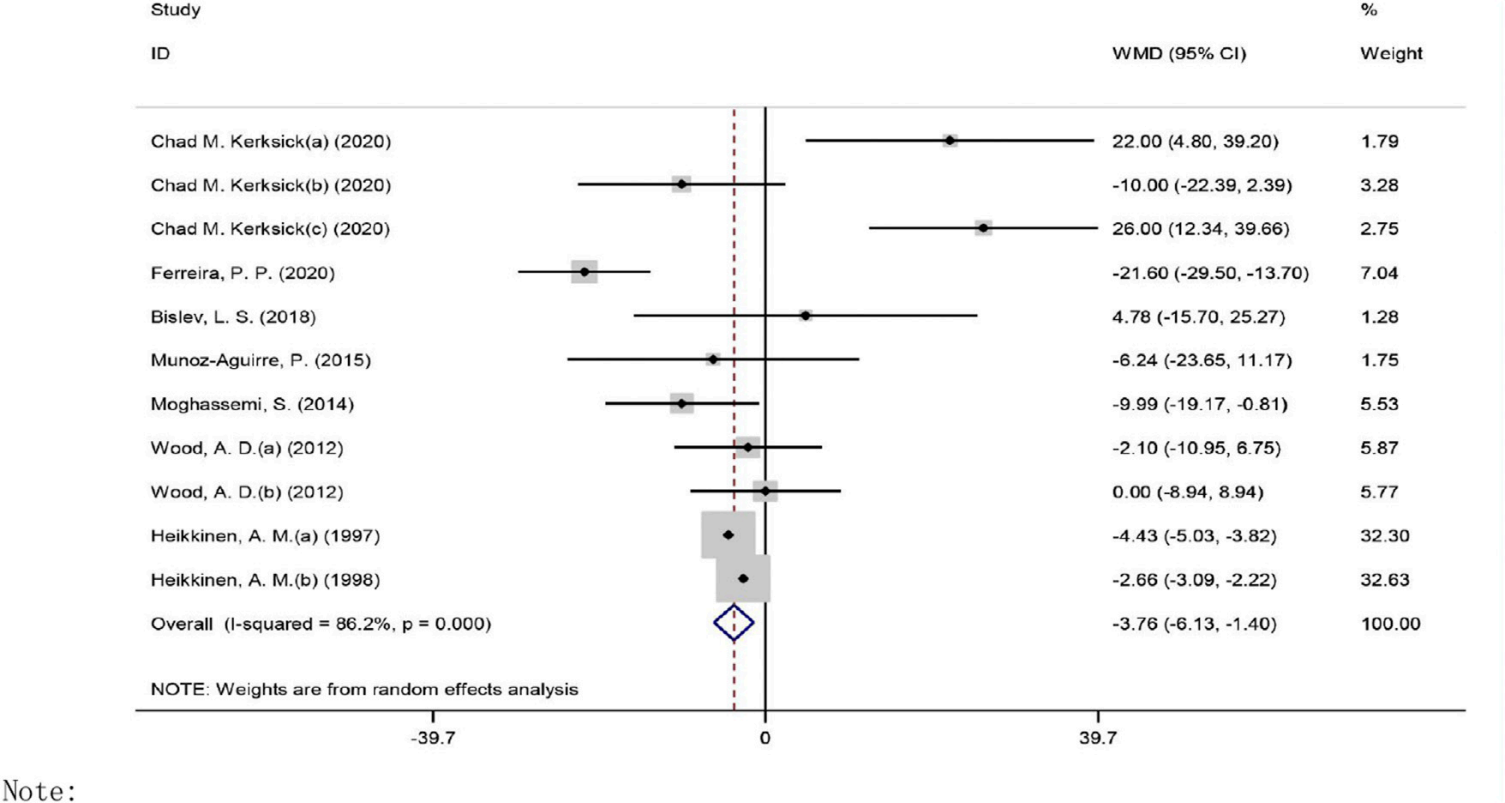
Forest plot of the randomized controlled trials investigating the effects of vitamin D on TG.

The overall effect of vitamin D intervention on TG level from 11 eligible trials (placebo = 505 participants, vitamin D intervention = 604 participants) are reported in Figure 4.

The administration of vitamin D led to a significant decrease of TG level at −3.76 mg/dl (CI: −6.12 to −1.39, *p* = 0.001), with a significant heterogeneity noted among the examined studies (I2 = 86%, *p* = 0.003). However, a notable reduction in TG concentrations was observed when vitamin D was prescribed in a dose of ≤400 IU/day (WMD: −2.42 mg/dl, 95% CI: −4.73 to −0.11, *p* = 0.039) *versus* ˃400 IU/day (WMD: −8.002 mg/dl, 95% CI: −17.88, 1.88, *p* = 0.113) (Supplementary Figure S1). In addition, a significant reduction in TG concentrations was detected in the participants with a BMI of 25.0–29.9 kg/m2 (WMD: −4.58 mg/dl, 95% CI: −6.66 to −2.49, *p* = 0.001) as compared to the participants with a BMI ≥30 kg/m2 (WMD: 7.84 mg/dl, 95% CI: −11.35, 27.05, *p* = 0.423). However, a notable decrease in TG levels was seen when vitamin D was administered for ≥26 weeks (WMD: −4.42 mg/dl, 95% CI: −6.52 to −2.32, *p* = 0.001) as compared to ˂26 weeks (WMD: 5.96 mg/dl, 95% CI: −10.07, 22.01, *p* = 0.464). Based on the results of the stratified analysis, there was a significant reduction of TG levels in the subjects with baseline TG concentrations ≥150 mg/dl (WMD: −15.95 mg/dl, 95% CI: −30.47 to −1.44, *p* = 0.031) *versus* ˂150 mg/dl (WMD: −2.59 mg/dl, 95% CI: −4.76to −0.42, *p* = 0.019).

#### Effects of Vitamin D on TC Levels

**FIGURE 5:**
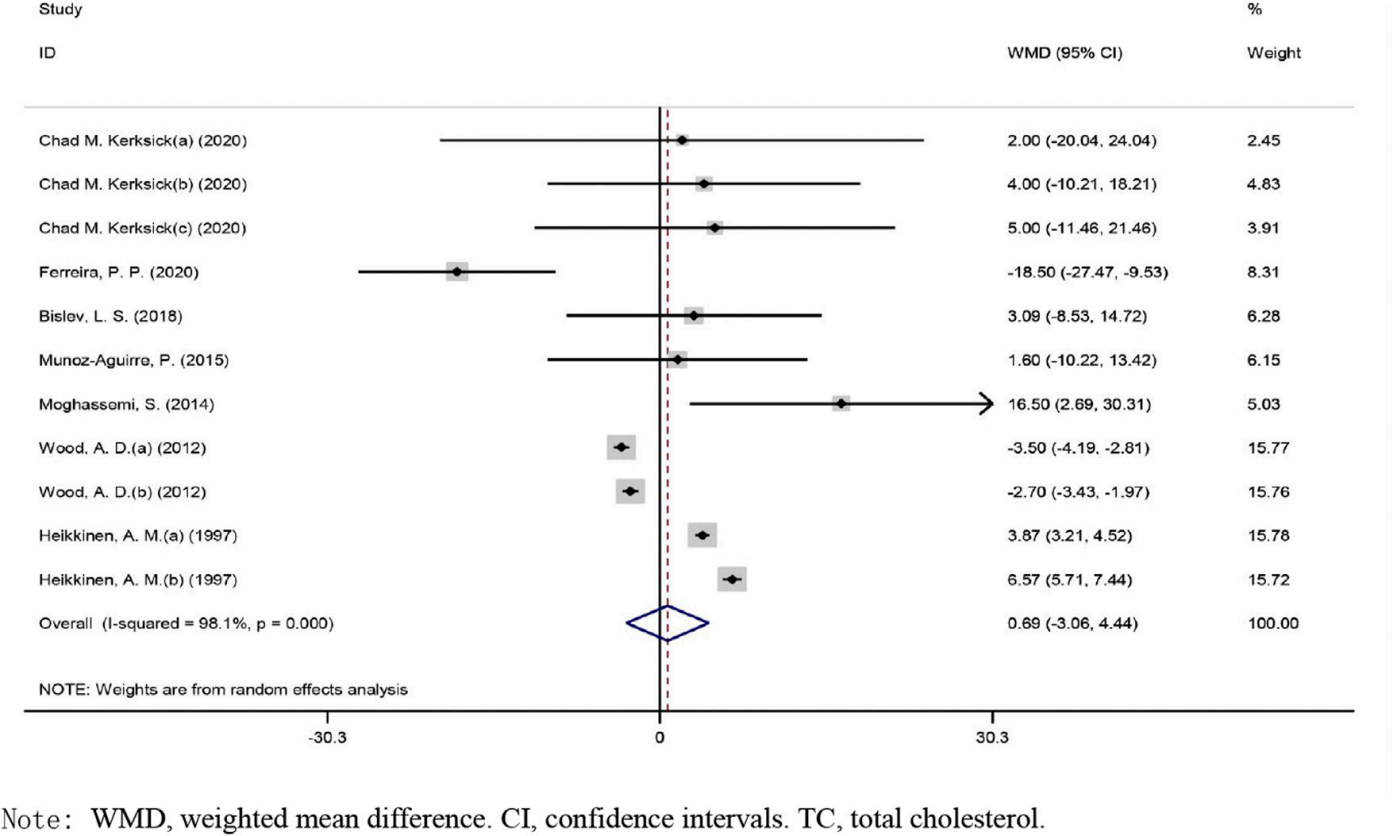
Forest plot of the randomized controlled trials investigating the effects of vitamin D.

The overall effect of vitamin D intervention on TC level from 11 eligible trials (placebo = 505 participants, vitamin D intervention = 604 participants) are reported in Figure 5. Vitamin D supplementation did not result in significant changes of TC concentrations (WMD: 0.689 mg/dl, CI: −3.059 to 4.438, *p* = 0.719), with a significant heterogeneity noted among the examined studies (I2 = 98%, *p* = 0.004). The results of the subgroup analyses did not reveal a significant impact of vitamin D administration on TC concentrations (Supplementary Figure S1).

## Sensitivity Analyses and Publication Bias

The sensitivity analyses were accomplished by sequentially eliminating each trial to evaluate the robustness of the overall results. The results of the present study were stable when a trial was eliminated and a significant change of the results did not happen (Supplementary Figure S2). No publication bias was found in the pooled effect sizes of LDL-C or TG levels in the funnel plots, as confirmed by Egger’s tests (Supplementary Figure S3). However, there was a significance publication bias for HDL-C and TC concentrations (Egger’s test: *p* = 0.042). The trim-and-fill sensitivity method was used to estimate the negative effect of unpublished studies and correct the effect of vitamin D intervention on HDL-C (−0.58 mg/dl, −0.99 to −0.17; *p* = 0.005, *n* = 16) and TC (−35.47 mg/dl, −43.29 to −27.65; *p* = 0.004, *n* = 36).

## Discussion

Our systematic review and meta-analysis demonstrate that the administration of vitamin D in postmenopausal women might lead to alterations of the lipid profiles, particularly in terms of TG and HDL-C reduction. However, based on our findings, the administration of vitamin D does not change TC or LDL-C levels in a significant manner. Moreover, it seems that the dose of vitamin D, the length of the intervention, the BMI of the participants and the baseline values for HDL-C and TG affected the effect of vitamin D on these parameters. To the best of our knowledge, this is the first systematic review and meta-analysis of RCTs that assessed the impact of vitamin D administration on serum lipids in postmenopausal women. A meta-analysis by Wang et al., in 2012 showed that the prescription of vitamin D led to an elevation in LDL-C concentrations in adults, but had no impact on HDL-C, TC or TG. In particular, LDL-C levels increased in the subjects who were diagnosed with obesity and when the intervention lasted for less than 12 months, whereas a statistically significant decrease in HDL-C was reported when the administration of vitamin D lasted for more than 12 months (Wang et al., 2012). However, Dibaba. (2019) revealed, in a recent meta-analysis based on data derived from 41 RCTs, that the administration of vitamin D decreased TG, TC and LDL-C, but had no impact on HDL-C. These results were found in the RCTs which lasted for less than 6 months and, for TG and LDL-C, in the subjects with a confirmed vitamin D deficiency (Dibaba 2019).

The post-menopausal period is associated with increased risks of cardiovascular disease, as the cardio-protective effect of estrogens is lost. Servadei et al. (2021) demonstrated that, in post-menopausal women, a tighter control of TG concentrations might prevent the development of atherosclerosis-related complications (Servadei et al., 2021). Thus, in this subgroup of patients, and in particular in post-menopausal females diagnosed with hypertriglyceridemia, the administration of vitamin D might provide significant benefits in terms of cardiovascular health, as our results demonstrate that the intervention was more effective in the subjects with baseline TG concentrations >150 mg/dl and when the administration of vitamin D exceeded 26 weeks. Interestingly, lower doses of vitamin D, namely less or equal to 400 UI/day, as well as the administration of vitamin D in overweight females, resulted in a more notable reduction of TG values. Jeenduang et al. (2019) reported that, during the post-menopause, vitamin D deficiency is associated with elevated TG levels, central obesity and the presence of the metabolic syndrome, and that females who suffer from vitamin D deficiency have elevated TG and TC concentrations, as well as an elevated waist circumference (Jeenduang et al., 2020). Thus, the decrease in TG values might have been related to the improvement in vitamin D serum status in the females with a possible undetermined vitamin D deficiency. In addition, there are studies that suggest that elevated TG concentrations are linked to several polymorphisms in the vitamin D receptor gene which may also explain why vitamin D was more effective in reducing TG in postmenopausal women at lower doses (Jin et al., 2021). On the other hand, we must take into consideration that the decrease of TG values was rather modest (−3.76 mg/dl) and it is unclear if it provides any benefits in terms of cardiovascular health. Thus, further validation of this finding in future RCTs/cohort studies is warranted.

The administration of vitamin D also impacted on HDL-C concentrations, i.e., vitamin D reduced HDL-C in postmenopausal females, particularly in overweight women, in women who had HDL-C levels within the normal range, when the vitamin D administration lasted for more than 26 weeks and when the vitamin D dosage exceeded 400 UI/day. Although HDL-C has been regarded as a divine messenger in reducing cardiovascular risk, recent studies show that cardiovascular events occur at a higher incidence in subjects with both low and high HDL-C concentrations. Thus, maintaining HDL-C levels within the normal range might be a better option in preventing the development of cardiovascular disorders during post-menopause (Barter and Genest 2019; Yu et al., 2020). However, Mirhosseini et al. (2018) et al., in their meta-analysis, concluded that vitamin D administration increases HDL-C and reduces LDL-C, TC and TG, and thus further research on this topic is needed (Mirhosseini et al., 2018). Moreover, it is noteworthy that the measurement of serum HDL-C might be inferior in terms of cardiovascular risk estimation to the assessment of HDL-C composition or function (Ben-Aicha et al., 2020).

It is important to take into consideration that, in the analyzed RCTs, vitamin D supplementation was prescribed to postmenopausal women with a normal status of health. Several other meta-analyses, conducted in subjects diagnosed with other illnesses, have provided promising results in terms of improvement of the lipid profiles following vitamin D administration. For example, Ostadmohammadi et al. (2019) highlighted in their meta-analysis of RCTs that vitamin D increased HDL-C concentrations in subjects with cardiovascular disorders (Ostadmohammadi et al., 2019). In addition, Miao et al. (2020), Jin et al. (2020) detected significant reductions in TC and LDL-C in females with polycystic ovary syndrome who were administered with vitamin D supplements (Jin et al., 2020; Miao et al., 2020). We suggested that postmenopausal women with an apparently normal status of health might not need to be adherent to vitamin D prescription regimen, as patients tend to prefer monthly to weekly or to daily administrations of vitamin D supplements (Rothen et al., 2020).

Our systematic review and meta-analysis has several strengths, but also some limitations. To our knowledge, this is the first systematic review and meta-analysis to analyze the impact of vitamin D supplementation on serum lipids in postmenopausal women. Moreover, since we only evaluated data derived from RCTs, the risk of bias was low. In addition, we conducted the systematic review and meta-analysis based on the PRISMA guidelines. Thus, the robustness of our results cannot be denied, particularly because we explored the sources of heterogeneity and we employed sensitivity analyses to clarify our findings. However, the number of analyzed RCTs and the study samples were low.

In conclusion, we show that vitamin D administration affects the lipid profiles in postmenopausal women. Further research, particularly well-designed RCTs with large number of patients will validate the findings of our systematic review and meta-analysis.
