# Supplementary material for: Vitamin D and Lipid Profiles in Postmenopausal Women: A Meta-Analysis and Systematic Review of Randomized Controlled Trials
Source: Front Mol Biosci. 2021 Dec 17;8:799934. doi: 10.3389/fmolb.2021.799934 (PMC8719197; doi:10.3389/fmolb.2021.799934)
Supplement: Supplementary file 1 [file Table1.docx]

**Supplementary Table 1: The risk of bias in studies included in this**

|  | Random Sequence Generation (selection bias) | Allocation concealment (selection bias) | Blinding of participants and personnel (performance bias) | Blinding of outcome assessment (detection bias) | Incomplete outcome data (attrition bias) | Selective reporting (reporting bias) | Other  bias | AHRQ standards |
| --- | --- | --- | --- | --- | --- | --- | --- | --- |
| Chad M. Kerksick.et  al.(a)(2020)( CTL = no diet + exercise control) |  |  |  |  |  |  |  | Good |
| Chad M. Kerksick.et  al.(b)(2020)( LCHC = low calorie high  carbohydrate diet) |  |  |  |  |  |  |  | Good |
| Chad M. Kerksick.et  al.(c)(2020)( LCHP = low calorie high  protein diet) |  |  |  |  |  |  |  | Good |
| Ferreira, P. P.et  al.(2020) |  |  |  |  |  |  |  | Good |
| Bislev, L. S.et al.(2018) |  |  |  |  |  |  |  | Good |
| Munoz-Aguirre, P.et al.(2015) |  |  |  |  |  |  |  | Poor |
| Moghassemi, S.et  al.(2014) |  |  |  |  |  |  |  | Good |
| Wood, A. D.et  al.(a)(2012) |  |  |  |  |  |  |  | Good |
| Wood, A. D.et  al.(b)(2012) |  |  |  |  |  |  |  | Good |
| Heikkinen, A. M.et al.(a)( 1997) |  |  |  |  |  |  |  | Good |
| Heikkinen, A. M.et al.(b)( 1997) |  |  |  |  |  |  |  | Good |
