# Supplementary figures and images for: Vitamin D and Lipid Profiles in Postmenopausal Women: A Meta-Analysis and Systematic Review of Randomized Controlled Trials
Source: Front Mol Biosci. 2021 Dec 17;8:799934. doi: 10.3389/fmolb.2021.799934 (PMC8719197; doi:10.3389/fmolb.2021.799934)

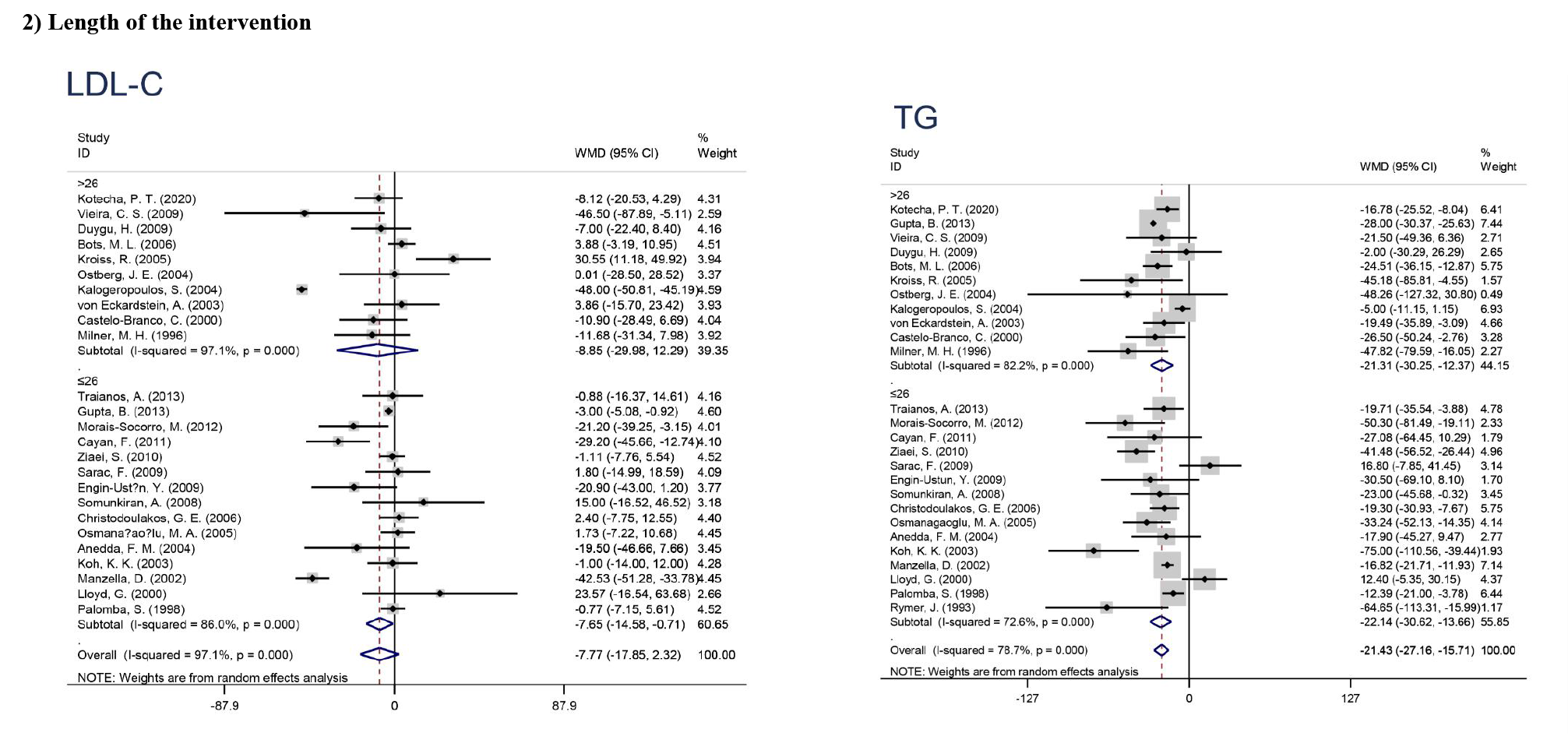

Supplement: Supplementary file 2 [file Image3.tif]

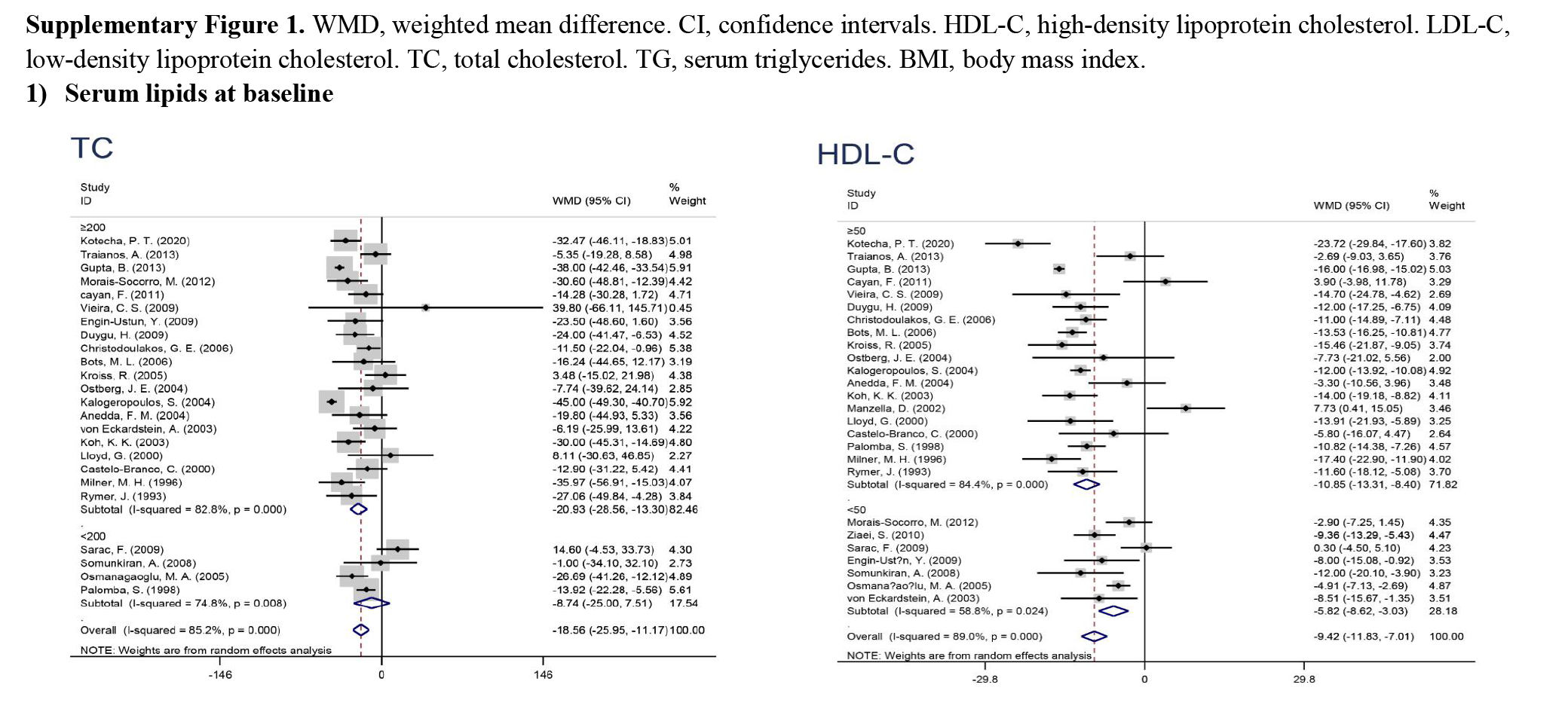

Supplement: Supplementary file 3 [file Image2.tif]

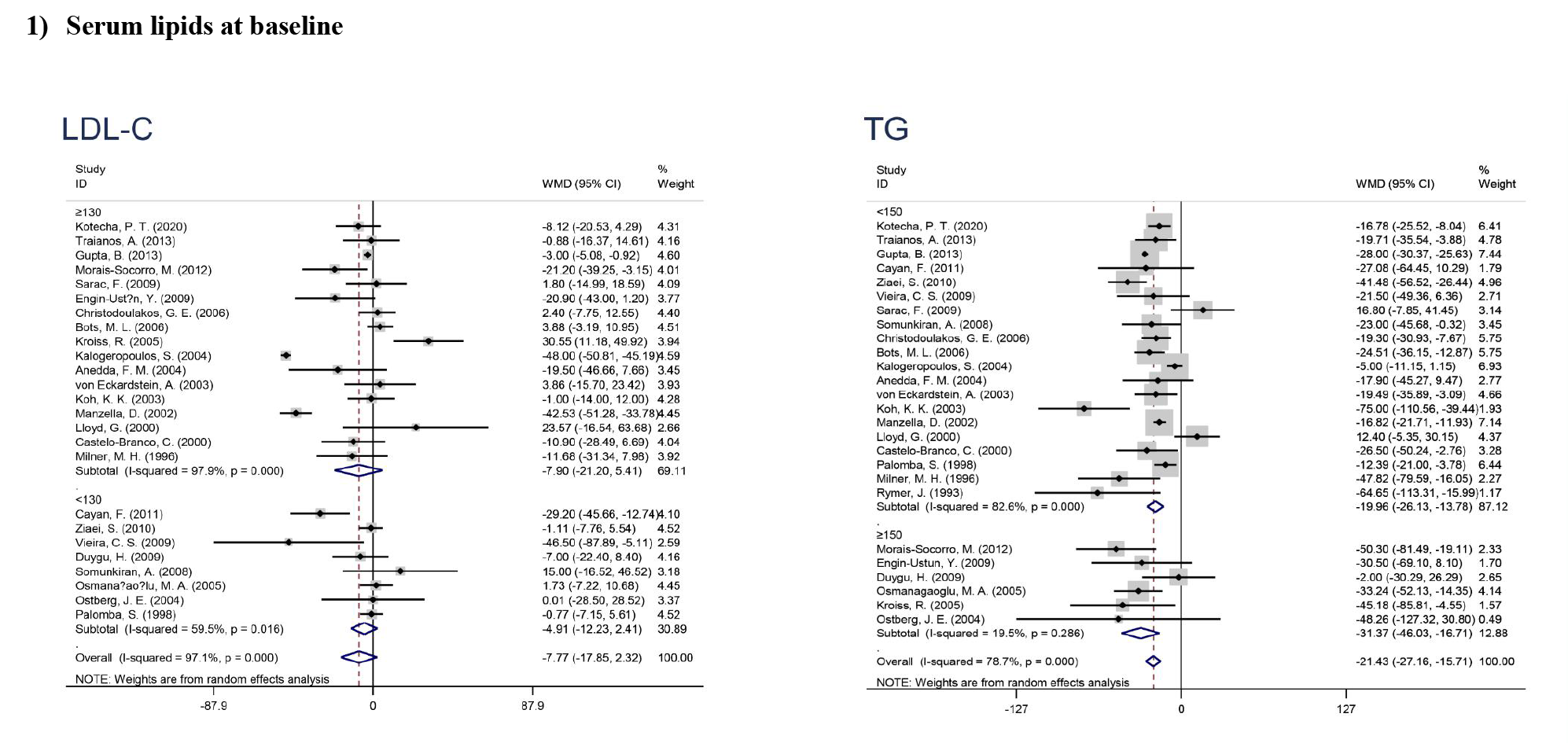

Supplement: Supplementary file 4 [file Image1.tif]
